# Discrepancy in Ratings of Shared Decision Making Between Patients and Health Professionals: A Cross Sectional Study in Mental Health Care

**DOI:** 10.3389/fpsyg.2020.00443

**Published:** 2020-03-24

**Authors:** Karin Drivenes, Vegard Ø. Haaland, Yina L. Hauge, John-Kåre Vederhus, Audun C. Irgens, Kristin Klemmetsby Solli, Hilde Regevik, Ragnhild S. Falk, Lars Tanum

**Affiliations:** ^1^Sørlandet Hospital, Kristiansand, Norway; ^2^Hospital Pharmacies Enterprise, South Eastern Norway, Oslo, Norway; ^3^Clinical Neuroscience Research Group, Department of Psychology, Faculty of Social Sciences, University of Oslo, Oslo, Norway; ^4^Oslo Centre for Biostatistics and Epidemiology, Faculty of Medicine, Institute of Basic Medical Sciences, University of Oslo, Oslo, Norway; ^5^Department of R&D in Mental Health, Akershus University Hospital, L renskog, Norway; ^6^OsloMet – Oslo Metropolitan University, Oslo, Norway; ^7^Norwegian Centre for Addiction Research, University of Oslo, Oslo, Norway; ^8^Vestfold Hospital Trust, Tønsberg, Norway

**Keywords:** SDM, mental health care, psychosis, user involvement, Collaborate, Shared decison-making

## Abstract

**Background:**

A defined goal in mental health care is to increase the opportunities for patients to more actively participate in their treatment. This goal includes integrating aspects of user empowerment and shared decision-making (SDM) into treatment courses. To achieve this goal, more knowledge is needed about how patients and therapists perceive this integration.

**Objective:**

To explore patient experiences of SDM, to describe differences between patient and therapist experiences, and to identify patient factors that might reduce SDM experiences for patients compared to the experiences of their therapists.

**Methods:**

This cross-sectional study included 992 patients that had appointments with 267 therapists at Sørlandet Hospital, Division of Mental Health during a 1-week period. Both patients and therapists completed the CollaboRATE questionnaire, which was used to rate SDM experiences. Patients reported demographic and treatment-related information. Therapists provided clinical information.

**Results:**

The analysis included 953 patient-therapist responder pairs that completed the CollaboRATE questionnaire. The mean SDM score was 80.7 (SD 20.8) among patients, and 86.6 (SD 12.1) among therapists. Females and patients that did not use medication for mental health disorders reported higher SDM scores than males and patients that used psychiatric medications (83.3 vs. 77.7; *p* < 0.001 and 82.6 vs. 79.8; *p* = 0.03, respectively). Patients with diagnoses involving psychotic symptoms reported lower SDM scores than all the other patients (66.8 vs. 82.3; *p* < 0.001). The probability that a patient would report lower SDM scores than their therapist was highest among patients that received involuntary treatment (OR 3.2, *p* = 0.02), patients with treatment durations longer than 2.2 years (OR 1.9, *p* = 0.001), and patients that required day care or in-patient care (OR 3.2, *p* = 0.01 and OR 3.2, *p* < 0.001, respectively).

**Conclusion:**

We showed that both therapists and patients reported good SDM experiences in decisional situations, which indicated that SDM was implemented well. However, the SDM scores reported by in-patients and patients with prolonged or involuntary treatments were significantly lower than scores reported by their therapists. Our findings suggested that it remains a struggle in mental health care to establish a common understanding between patients and therapists in decisional processes regarding treatments for some patient groups.

## Introduction

In the past few decades, awareness has been raised among mental health professionals, politicians, patient organizations, and health administrators regarding the advantages of patients playing a more active role in their own treatments. This approach entails a shift for health services from focusing on treatments to focusing on patients (Shultz, 1985). Shared decision-making (SDM) is becoming part of modern health care worldwide (Slade, 2017), and it should preferably be integrated into all treatment programs, including mental health care and interdisciplinary specialized addiction services (Swanson et al., 2007; Huang et al., 2019).

A variety of definitions for SDM have been suggested since the concept was introduced in the 1990’s (Charles et al., 1999). The most common definition was given by Glen Elwyn: “An approach where clinicians and patients share the best available evidence when faced with the task of making decisions, and where patients are supported in considering options to achieve informed preferences” (Elwyn et al., 2010). A crucial prerequisite for SDM is that the perspectives of patients and therapists are equally valued, despite fundamental differences (Makoul and Clayman, 2006). The therapists hold a professional expertise based on education and clinical practice, whereas the patients hold an expertise from the experience of managing a life with illness.

Traditionally, SDM was understood as a micro-social process, limited to a single consultation involving the patient and therapist. Morant et al. (2016) suggested that this understanding of SDM was too narrow and limited for mental health care. Their main objection rested on the nature of mental illness and its demand for complex management. Mental health care most often includes long-term treatment that includes key players, like relatives and people in the patient’s supportive network. Additionally, it is important to recognize that mental illnesses evolve through periods of recovery and relapse (Perestelo-Perez et al., 2011; Beitinger et al., 2014; Morant et al., 2016). All these elements call for considering SDM in mental health care a continuous process that involves multiple people, which cannot be restricted to a single decision or a particular consultation (Chong et al., 2013a; Kilpatrick et al., 2019).

To develop the mental health care service further, more knowledge is needed about the ability of our health service to include patient perspectives in treatment situations. Previous studies that aimed to evaluate SDM implementations in mental health care services were often restricted to specific clinical settings or diagnostic groups (Hamann et al., 2007; Forcino et al., 2018; Kayyali et al., 2018; Inder et al., 2019), or alternatively, they mainly focused on describing the patient benefits provided with SDM education (Hamann et al., 2006; Duncan et al., 2010; Slade, 2017). In those studies, more positive SDM experiences were associated with older patient ages and female gender (Forcino et al., 2018). However, we lack knowledge about how a general population in a specialist mental health care setting experiences SDM (Elwyn et al., 2013).

A number of tools for assessing SDM have been developed and validated. These tools include interviews, paper-based self-report forms, interactive voice-response calls, or questionnaires conducted on a tablet or computer (Makoul and Clayman, 2006; Bouniols et al., 2016; Kienlin et al., 2017). SDM assessments can be conducted on site or retrospectively (Barr et al., 2017). However, in many publications, it is not clear which assessment tools were used to measure SDM experiences (Stovell et al., 2016).

Shared decision-making implementation requires contributions from individuals with different perspectives; consequently, disagreements can occur. Previous explorations of the nature of patient-therapist relationships have indicated an existence of non-independency, as the clinical encounters contain multiple persons embedded within a social context (Kenny et al., 2006). Such a context includes interpersonal relationships, suggesting the individual experiences of the participants mutually reinforcing and non-independent of each other (Petrocchi et al., 2019). We suggest that a higher level of agreement on how to perform decisional processes represents a better foundation for good treatment decisions, which facilitate patient compliance. Although the level of agreement is rarely studied, psychotherapy studies have suggested that greater agreement on the quality of the patient-therapist alliance and stronger patient-therapist bonds could lead to better treatment outcomes (Laws et al., 2017; Rubel et al., 2018). A discrepancy can occur between the patient and therapist experiences in SDM, when either the therapist or the patient experiences a better SDM process. A study that explored the fit between patient and therapist orientations suggested that a better fit could improve patient satisfaction, but that the patient’s orientation was more important to patient satisfaction than the therapist’s orientation (Krupat et al., 2000). A large discrepancy might indicate that the patient and therapist do not share or communicate common goals or that they do not have a similar appreciation of the usefulness of the treatment. From the patient’s perspective, it is irrelevant whether the therapist’s experience is worse than the patient’s experience in SDM, but when the patient’s experience is worse than the therapist’s experience, it might hinder an optimal decision process. Additionally, we hold the health care service responsible for facilitating patient involvement in decisions regarding their health and treatment. Therefore, in this study, we chose to focus on the fraction of patients that experienced more negative SDM processes than their therapists, because this situation was suggested to be more important for good treatment courses and outcomes (Krupat et al., 2000). In this study, we termed this situation “a negative discrepancy.”

## Materials and Methods

### Aims

The aims of this study were to (*i*) explore patient experiences with SDM in a mental health care and addiction service setting, (*ii*) describe the congruence in SDM experiences between patients and their therapists; and (*iii*) identify factors associated with more negative SDM experiences for the patient than for their therapist (a negative discrepancy).

### Population

Sørlandet Hospital Trust serves a population of about 300 000 individuals (Statistics Norway, 2019) in the southern part of Norway. It provides medical health services to a number of rural and urban communities. The Division of Mental Health and Addiction Services has 280 beds, and it provides acute and long-term treatments in forensic psychiatry, child and adolescent psychiatry, geriatric psychiatry, and treatment of substance-related disorders. The division has 1375 full-time-equivalent employees, and it manages 4150 admissions and 184 000 consultations per year.

### Data Collection

We recruited patients that received psychiatric health care at the hospital during the third week of January 2017. Patients over 16 years were included. Day care patients and out-patients were included from visit 2 when they arrived at the clinics for their regular appointment. Patients receiving ambulatory care (treatment offered at the patients’ residents), were included during the visit from health care professionals. In-patients were included at a scheduled talk after 24 h of hospitalization. Both patients receiving involuntary treatment were also included. Patients were only included at the first visit, when they had more than one visit or contact with the service during the week. Patients that received treatment from different parts of the division within the study week were only included once. Patients without legal capacity to make informed consent were not asked to participate. Patients were excluded, when for any reason, participation was contraindicated by their therapist, they could not complete a paper-based questionnaire, or they could not read the Norwegian language. All patients provided written informed consent after receiving oral and written information about the study from their therapist, from posters in the clinic, and/or from study personnel.

The assessment consisted of two parts: one part was completed by the patient, and the other part was completed by their therapist. All patients completed their part of the inventory during a visit at the clinic. The patients completed the CollaboRATE measurement tool and a form with questions about demographic characteristics and medication use. On the same day, the patient’s therapist also completed the CollaboRATE and a form with questions about the patient’s diagnoses and clinical characteristics.

### Research Instrument

We based all SDM outcomes on the CollaboRATE measurement tool. The CollaboRATE is a well validated, self-reporting, paper assessment tool that was shown to be useful in different patient populations and at different levels of care (Forcino et al., 2018). The CollaboRATE was developed to accommodate both patient and therapist experiences (Elwyn et al., 2012). It comprised three single questions related to education about the health situation, and whether professionals paid attention to what matters most to the patient. Questions were rated on a scale of 0–9, where zero represented “no effort was made” and nine represented “every effort was made” (Barr et al., 2014). The responses from the three questions were summed, and the range of total scores was 0–27. According to the CollaboRATE manual, this sum score was multiplied by 3.704 to convert it to a response percentage score that ranged from 0 to 100%. We were aware of ceiling effects with patient-reported SDM (Makoul et al., 2007; Barr et al., 2014; Forcino et al., 2018), therefore, we also reported the proportion of top scores (score = 100).

### SDM Dyadic Deviation Value

The different experiences with SDM were explored by calculating a SDM dyadic deviation value. This calculation was the patient’s recalculated CollaboRATE percentage score minus the therapist’s corresponding score. The result was positive, when the patient reported a higher SDM score than the therapist, and it was negative, when the patient reported a lower SDM score than the therapist. A larger absolute SDM dyadic deviation value indicated a larger difference between patient and therapist SDM experiences. A SDM dyadic deviation value of zero reflected situations where the patient and therapist reported the same CollaboRATE scores.

According to a consensus between the expert group that initiated the study and clinicians experienced in the field, a clinically relevant negative difference between patient and therapist experiences was defined by a cut off value set to −22 on the CollaboRATE (range 0–100). When the negative discrepancy was −22 or lower, the patients reported at least 6 points less than their therapist on the CollaboRATE ordinal scale (range 0–27), which represented about a 20% difference.

Patient-therapist pairs with a negative discrepancy of −22 or lower were designated group one. Patient-therapist pairs with a SDM dyadic deviation value higher than −22 were considered to have corresponding experiences, and they were designated group two. Thus, group two contained pairs with SDM dyadic deviation values close to zero or in the positive range. We considered whether it would be appropriate to include patient-therapist pairs with SDM dyadic deviation values of −22 to 22 in the same group as those with SDM dyadic deviation values >22 in the model. To that end, we also analyzed patients with SDM dyadic deviation values >22 as a separate group. This alternative model produced the same results as those produced with the chosen model, for the available variables.

### Ethical Approval

The study was approved by the Norwegian Regional Committee for Research Ethics (no. 2016/1781) and the Hospital Research Board (no. 17/00104). All patients provided written informed consent prior to participation.

### Data Analysis

Patient diagnoses were categorized into diagnostic groups, according to the International Classification of Diseases, tenth revision (ICD-10) (World Health Organization [WHO], 2017). The diagnostic groups were classified as follows (with shortened group names in parentheses): F10 (Substance related disorders); F20 plus the F30 subgroups F30.1, F30.2, F30.8, F30.9, F31.1, F31.2, F31.5, F32.3, and F33.3 (Psychotic disorders); all other F30 subgroups (Affective disorders); F40 (Anxiety disorders); F60 (Personality disorders); and F90 (Behavioral disorders). Patients with other main diagnoses were combined into a group called “Other” (Table 1).

**TABLE 1:** Characteristics of the patients, CollaboRATE mean scores and proportion top scores for the different subgroups of patients, and statistical significance of differences between the subgroups, *n* = 992.

Patient characteristics	*N* (%)	Mean (SD)	CollaboRATE mean score (SD)^a^	*p*-value	CollaboRATE top score *N* (%)
Total	992		80.7 (20.8)		272 (27.4)
Age, years		35.6 (13.2)			
**Gender**					
Female	575 (58.0)		83.3 (20.1)	<0.001	186 (32.3)
Male	417 (42.0)		77.7 (21.0)		86 (20.6)
**Medication for mental health concern**					
Yes	567 (57.2)		79.8 (21.4)	0.03	144 (25.4)
No	425 (42.8)		82.6 (19.5)		128 (30.1)
**Main diagnostic group**					
F10 Substance related disorders	187 (18.9)		79.1 (24.1)		60 (32.1)
F20, F30.1, F30.2, F30.8, F30.9, F31.1, F31.2, F31.5, F32.3, F33.3 Psychotic disorders	82 (8.3)		66.8 (25.1)	<0.001^b^	9 (11.0)
The remaining F30 Affective disorders	192 (19.4)		84.4 (18.7)		60 (31.3)
F40 Anxiety disorders	285 (28.7)		85.0 (17.4)		90 (31.6)
F60 Personality disorders	75 (7.6)		76.7 (21.1)		16 (21.3)
F90 Behavioral disorders	48 (4.8)		85.8 (15.7)		15 (31.3)
Other diagnosis or missing information	123 (12.4)		82.0 (16.8)		22 (17.9)
**Involuntary treatment**					
Yes	30 (3.0)		50.6 (29.6)	<0.001	2 (6.6)
No	962 (97.0)		82.0 (19.6)		270 (28.1)
**Treatment duration**					
Mean, years		5.2 (6.7)			
≤median 2.2 years	496 (50.0)		83.9 (18.1)	<0.001	147 (29.6)
>median 2.2 years	496 (50.0)		78.0 (22.5)		125 (25.2)
**Level of care**					
In-patient care	106 (10.7)		70.3 (26.3)		20 (18.9)
Ambulatory care	73 (7.4)		73.1 (22.7)		8 (11.0)
Day care	38 (3.8)		72.4 (24.7)		8 (21.1)
Out-patient care	761 (76.7)		83.8 (18.3)	<0.001^c^	232 (30.5)
Missing information	9 (0.9)				

*^a^n = 956 patients completed the CollaboRATE. ^b^Compared to the patients with the remaining diagnoses (with mean CollaboRATE score 82.3). ^c^Compared to the patients at the remaining levels of care (with mean CollaboRATE score 71.9).*

Age was considered a continuous variable. Gender, use of psychotropic medication (yes/no), and involuntary treatment (yes/no) were dichotomized. Treatment duration was dichotomized, as greater or less than 2.2 years, which corresponded to the median treatment duration. We could not retain treatment duration as a continuous variable, because it was not linearly related to the dependent variable. The levels of care were categorized into four groups: in-patients, day care, ambulatory care, and out-patients.

Statistical analyses were performed with the Statistical Package for the Social Sciences (SPSS), developed by IBM Corporation, 23rd edition (IBM SPSS Statistics 23) and Stata Statistical Software (Stata) Release 15 (STATA, 2019). Patient characteristics were compared with the independent *t*-test and chi-square test. Variables that described the SDM experience for patients and therapists are expressed as the frequency, proportion, or the mean and standard deviation (SD).

To take into account the non-independence in the data, we performed mixed effect logistic regression analyses to identity variables that influenced the SDM dyadic deviation value. Then, patients belonging to the same therapist were grouped together, and dependencies within therapist were estimated by including a random effect to the model. We used the purposeful selection approach to select variables for these analyses (Altman, 1991). First, we performed univariate analyses with the following variables: age, gender, diagnosis, level of care, involuntary treatment, drug treatment, and treatment duration. Variables with a *p*-value < 0.2 were included in the multivariate analyses. In the multivariate analyses, variables with the largest *p*-values were deleted one-by-one, until all variables were significant at the 5% level. Results are presented as the odds ratio (OR) with 95% confidence interval (CI). No effect of multi-collinearity was observed, because all variance inflation factors were <2.

We also performed a sensitivity analysis to study the sensitivity of the chosen cut-off value of −22 for the reported SDM. We repeated mixed effect logistic regression analyses with cut-off values of −18 and −26, which represented patient scores that were five and seven points less than the therapist scores, respectively.

An intra-class correlation coefficient (ICC) (Altman, 1991) was calculated to identify correlations between patient and therapist SDM scores. Spearman’s rho correlation coefficient was calculated to evaluate the association between the patient’s SDM score and the SDM dyadic deviation value.

## Results

### Patient Characteristics

We included 992 patients with a mean age of 35.6 years, and 58% were female (*n* = 575). Of these, 567 (57%) received medications for treatment. Anxiety disorders were the most common diagnosis (*n* = 285 patients, 28.7%), followed by affective disorders (*n* = 192 patients, 19.4%), substance-related disorders (*n* = 187 patients, 18.9%), and psychotic disorders (*n* = 82 patients, 8.3%). Involuntary treatment was established for 30 patients (3.0%). Most patients (*n* = 761, 76.7%) received out-patient care, and 106 (10.7%) received in-patient care. The mean treatment duration for all patients was 5.2 years, and the median treatment duration was 2.2 years (Table 1).

The 267 therapists that completed the therapist parts treated a mean of 3.7 patients (range 1–22). Not all patients completed the CollaboRATE. The final SDM exploration included 953 patient-therapist responder pairs.

### Experiences With SDM

The patient CollaboRATE reports showed a mean SDM score of 80.7 (SD 20.8; Table 1). Male patients reported a significantly lower SDM score than females (mean 77.7 and 83.3, respectively; *p* < 0.001). Patients that used medication for mental health concerns (*n* = 567) reported significantly lower SDM scores than patients that did not use medication (mean SDM scores: 79.8 and 82.6, respectively; *p* = 0.03). The 82 patients with psychotic disorders reported significantly lower SDM scores than patients without psychotic disorders (mean SDM scores: 66.8 and 82.3, respectively; *p* < 0.001). Patients treated involuntarily (*n* = 30) reported significantly lower SDM scores than patients treated voluntarily (mean SDM scores: 50.6 and 82.0, respectively; *p* < 0.001). Additionally, patients with treatment durations longer than the median of 2.2 years reported significantly lower SDM scores than patients with shorter treatment durations (mean SDM scores: 78.0 and 83.9, respectively; *p* < 0.001). Out-patients (*n* = 761) reported significantly higher SDM scores than patients that received other levels of mental health care (mean SDM scores: 83.8 and 71.9, respectively; *p* < 0.001).

The top SDM score was reported by 272 patients (27.4%), more frequently by females than by males (32.3 and 20.6%, respectively; *p* < 0.001). Top SDM scores were also frequently reported by patients that received out-patient treatments (30.5%, *p* < 0.001). In contrast, top SDM scores were reported less frequently by patients that received ambulatory and in-patient care (11.0%, *p* = 0.001, and 18.9%, *p* = 0.04, respectively). The proportion of top scores among patients that received day care was not significantly different from those reported by patients that received other treatment levels. Top SDM scores were reported by only nine out of 82 patients (11.0%) with psychotic disorders. In contrast, 261 out of 910 patients in the other diagnosis groups (28.6%) reported top SDM scores (*p* < 0.001). No significant differences were found among the other diagnostic groups (data not shown).

The mean SDM score for therapists was 86.6 (SD 12.1), and a top score was reported by therapists for 188 patients (19.7%; data not shown).

### Distribution of SDM Dyadic Deviation Values

**FIGURE 1:**
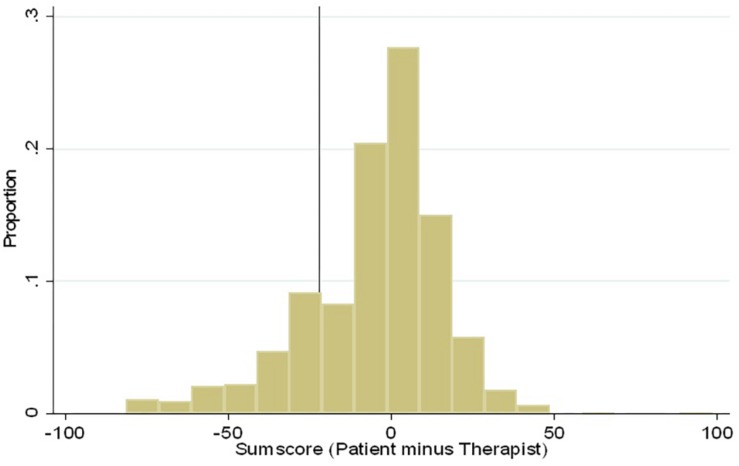
Distribution of SDM dyadic deviation values among the patient-therapist pairs in the study *n* = 953.

The mean SDM dyadic deviation value was -5.8 (SD 20.9, range: −82 to 96). The distribution of SDM dyadic deviation values are shown in Figure 1. Group one (SDM dyadic deviation values ≤ −22) contained 192 patient-therapist pairs (20%); group two (SDM dyadic deviation values >4−22) contained 761 pairs (80%). Among the patient-therapist pairs in group two, 703 pairs (74%) had a SDM dyadic deviation value between −22 and 22; 58 pairs (6%) had a SDM dyadic deviation value >22.

### Factors Associated With SDM Dyadic Deviation Values

Patients that received in-patient care or day care were more than three-fold more likely of being in group one (having a negative discrepancy) compared to patients that received out-patient care (OR 3.2, 95% CI: 1.7-6.0 and OR 3.2, 95% CI: 1.3–8.0, respectively; Table 2). Patients that received involuntary treatment also had more than three-fold higher risk (OR 3.2, 95%CI: 1.2–8.5) of being in group one compared to patients that received voluntary treatment. Additionally, patients that had been in treatment for more than 2.2 years had a 1.9-fold higher risk (95% CI: 1.3–2.8) of being in group one compared to patients treated for less than 2.2 years. Patient age, gender, diagnosis, and use of medication for a mental health disorder did not reach statistical significance, and thus, not considered associated with a negative SDM dyadic deviation value.

**TABLE 2:** Variables associated with lower SDM dyadic deviation values. Results from mixed effect logistic regression analyses.

	Univariable analysis OR (95% CI)	*P*-value	Multivariable analysis^a^ OR (95% CI)	*P*-value
**Age (per 10 years)**	1.0 (0.9–1.2)	0.57	–	
**Gender**			–	
Female	0.9 (0.6–1.2)	0.40		
Male	Ref			
**Medication for mental health concern**			–	
Yes	1.3 (0.9–1.9)	0.16		
No	Ref			
**Psychotic disorders ^b^**			–	
Yes	4.0 (2.1–7.7)	<0.001		
No	Ref			
**Involuntary treatment**				
Yes	6.6 (2.7–16.1)	<0.001	3.2 (1.2–8.5)	0.02
No	Ref		Ref	
**Treatment duration**				
≥2.2 years	2.4 (1.6–3.5)	<0.001	1.9 (1.3–2.8)	0.001
<2.2 years	Ref		Ref	
**Level of care**				
In-patient	4.3 (2.3–7.8)	<0.001	3.2 (1.7–6.0)	<0.001
Ambulatory care	3.0 (1.5–6.1)	0.002	2.1 (1.0–4.3)	0.06
Day care	3.7 (1.5–9.2)	0.005	3.2 (1.3–8.0)	0.01
Out-patient	Ref		Ref	

*OR, odds ratio; CI, confidence interval. ^a^n = 943 observations with complete information about all variables in the multivariable model. ^b^Includes the ICD-10 diagnoses F20, F30.1, F30.2, F30.8, F30.9, F31.1, F31.2, F31.5, F32.3, and F33.3.*

The correlation coefficient between patient SDM experiences and the SDM dyadic deviation values was 0.83, which indicated a strong positive relationship. The sensitivity analyses showed that cut-off values of −18 and −26 produced results similar to those produced with a cut-off of −22. The effects of covariates showed ORs similar to those obtained with the −22 cut-off value in the original model (data not shown).

## Discussion

Both patients and therapists reported average CollaboRATE scores greater than 80 out of 100. This suggested that the SDM experiences were good in decision situations and that SDM was generally well-implemented in the hospital. SDM experiences were less successful among patients in need of higher levels of care, patients that used medication for mental health concerns, patients that received involuntary treatments, and patients that required prolonged treatments. These groups of patients were most likely to report negative SDM dyadic deviation values.

Our findings on patient SDM experiences were comparable to those previously reported by Forcino et al. (2018) in primary care, where the mean SDM varied from 68 to 86 out of 100. In both studies, patients with psychotic disorders or involuntary treatments reported more negative experiences with SDM than patients with other diagnoses. In our study, men reported more negative SDM experiences than women, also consistent with findings from Forcino et al. (2018).

From the clinical perspective, patient groups that reported lower SDM scores more often had serious mental illnesses that demanded more comprehensive treatments, including long-term medications. Although not included in the present model, the severity of the disorder was likely to be correlated with the SDM score. Moreover, the implementation of SDM might be more difficult in these patient groups, due to multiple factors regarding treatments. These speculations are consistent with findings from a Swedish study performed by Rosenberg et al. (2017), where patients with serious mental illnesses in municipal social psychiatry units reported variable SDM experiences.

Three quarters of our patient-therapist pairs reported similar SDM experiences. However, among one fourth of the pairs, patient SDM experiences were clearly different from those of the corresponding therapists. This discrepancy in experiences was suggested to be due to deficits among both patients and the therapists (LeBlanc et al., 2009). A recent study that explored the therapeutic bond in mental health care services suggested that, when both the patient and therapist of a dyad perceived similar changes in the therapeutic bond, they worked more effectively toward symptom improvements (Rubel et al., 2018). Additionally, the study from Rosenberg and co-workers suggested that the patient-therapist relationship was a key factor in facilitating SDM. However, the present study also included clinical and structural factors of the service; therefore, the discrepancy in SDM experiences might have been an expression of shortages on levels other than those explored in the study. The shortages may be patient-related, like opposition to the diagnosis or a wish to attend other treatment courses than offered. Shortages may also be related to the health care service, like therapist availability or practical organization of the treatments.

Based on findings from previous studies (Farrelly et al., 2015; Grim et al., 2016), it was not surprising that patients that received involuntarily treatments, prolonged treatments, or required more intensive care reported the lowest SDM scores. Nevertheless, it has been suggested that the application of SDM was feasible and beneficial for these groups of patients (Hamann et al., 2003; Shay and Lafata, 2015). Therefore, it was not quite clear why these patient experiences differed from the experiences of their therapists. We suggest that these findings might be contextually linked to the structure and/or framework of the service. Some treatment levels might not facilitate SDM implementation, due to a strict framework or lack of alternative treatments; thus, disagreements between patients and therapists might be more likely to occur in these circumstances. Indeed, treatments for in-patients include many predetermined factors that cannot be altered to meet an individual patient’s needs and preferences. The same caveat applies to the remaining treatment levels. Structural frameworks, like house rules in in-patient clinics and attendance times in other clinics, can restrict the range of possible adjustments. Another explanation might be that some patients lacked sufficient competence to participate in SDM, due to a serious debilitating mental illness or an impaired ability to modulate emotions or understand their mental health prognosis (Chong et al., 2013b).

Although patients that received involuntary treatments had a higher probability of reporting more negative SDM scores than their therapists, we would like to emphasize that the service has the responsibility of actively including these patients in decisions, when possible. We suggest that it is particularly demanding to establish SDM among patients treated involuntarily, due to the fundamental difference in understanding. The patients treated involuntarily might have evaluated their health situation differently from how the health care service evaluated it, and thus, they might not agree to arranged treatments. However, the negative SDM scores cannot be explained by the involuntary situation alone. A recent study revealed that patients treated involuntarily identified involvement in clinical decisions as a key factor in improving their experience of care (Burn et al., 2019). We suggest these patients could be involved in some treatment options, and that therapists should be aware of and utilize those opportunities. Continuous efforts to facilitate SDM for patients treated involuntarily should be encouraged.

Difficulties in establishing an effective treatment might lead to a poor SDM experience for the patient. These difficulties might be caused by an ineffective treatment, side effects from medications, persistent delusional symptoms, or unrealistic treatment goals. Additionally, negative SDM dyadic deviation values might be due to a basic discrepancy in understanding the situation between the patient and the therapist. For example, the therapist might feel that sufficient effort has been devoted to treatment and that the available treatment options have been explored, while the patient conceives it differently. A previous study suggested that the therapeutic atmosphere might change over time during long-term treatments (Sauer et al., 2003). Patients just starting treatment might have a more positive SDM experience than patients that have been undergoing treatment for a long time, due to a fundamental shift in their understanding of the therapeutic benefit of the treatment. Moreover, the treatment atmosphere reflected in a negative SDM dyadic deviation value might affect the success of the treatment.

Patients that received short-term out-patient treatments reported higher SDM scores, they were less likely to report negative SDM dyadic deviation values, and they more frequently reported top SDM scores, compared to patients in other levels of care. These treatment profiles indicated that patients with less severe disorders, and perhaps, less distress had better SDM experiences. Hence, parts of the service treating other patient groups might adopt some treatment approaches that facilitate SDM. Despite the different premises for different treatments, there might be advantageous treatment elements that could be implemented in other service areas, where patients reported low SDM scores.

In the statistical analyses, the SDM dyadic deviation values were dichotomized into two groups. Patients that clearly reported a more positive SDM than their therapist was an interesting subgroup, but we found no differences in the variables between this group and the group of patients with SDM experiences similar to their therapists. Thus, these two subgroups of patients were considered one group in the statistical analyses. We speculated that, if the SDM dyadic deviation values in the positive range were spaced into a separate group, then we might have detected other differences between the groups. However, we did not make such findings as we tested the model. In addition, if we had had access to data on more variables, we might have been able to reveal associations specific for patients with positive SDM dyadic deviation values.

### Strengths and Limitations

In Norway, both primary care and specialist care are mainly publicly operated. Therefore, we considered the patients in this study representative of patients that received mental health care at the specialist level in Norway. This was a major strength of the study. However, we could not determine whether all the characteristics of the included patients were representative of the national population with mental illnesses, because that information would be very difficult to acquire (Øy, 2019). Nevertheless, due to the relatively high number of participants in our study, we suggest that our findings were relevant to the understanding of the congruence between patient and therapist experiences in mental health care at the specialist level.

Another strength of this study was the high proportion of completed CollaboRATE questionnaires; thus, we retained nine tenths of the included cases in the analyses. The CollaboRATE questionnaire was a suitable tool for revealing the extent of SDM in specialist practice. Nevertheless, although SDM has been implemented for some time in many institutions, we lack a common definition for “sufficient SDM”; moreover, we lack a common set of attitudes or skills that should be applied when exploring these processes. Consequently, the definition of “sufficient SDM” should be explored further and discussed in future studies to establish a consensus on what comprises “sufficient SDM”.

Patients in need of more complex care interact with more professionals during treatment. They meet one or more therapists for active treatment sessions, and other personnel for activity groups and other arrangements. Thus, the SDM reports from these patients could potentially reflect decision processes and decisions that involved professionals other than the main therapist. However, in the present study, only the main therapist provided the other half of the dyadic SDM value. Therefore, the SDM reports from the patients, and thereby the dyadic SMD value, might not have reflected strictly the relationship between two reporting individuals. Due to our knowledge of the guidelines and management of our division, we knew that patients with serious mental illnesses were more likely to receive long-term treatment and more frequent follow-ups at the specialist care level. However, we did not request any assessment of illness severity or level function in addition to diagnostic information. Due to the variability in illness severity within each diagnostic group, it might have been advantageous to record the current illness severity, to get a wider foundation to understand patient situations. No information about comorbidity was available for analyses, which was a limitation preventing us to explore the patient situation in a more comprehensive way.

Another limitation of this study was that the analyses of different independent variables led to several subgroups with very few patients. This limitation might have precluded the detection of significant differences between certain groups. Although we found a positive relationship between lower patient-reported SDM scores and a negative SDM dyadic deviation value, we did not find associations between the SDM dyadic deviation value and gender, medication use, or a psychotic disorder diagnosis among the patients in group one. This lack of associations may probably be due to low patient numbers in each subgroup in the statistical model. However, merging different subgroups was not considered correct from a clinical standpoint, due to the diversity between groups. Finally, factors other than those explored in the present study might, presumably, have influenced the SDM experiences.

### Implications

The findings of this study indicated that the perceptions of SDM were generally high in the specialist mental health services in Norway, but differed between patients and their therapists. Moreover, these patient-therapist discrepancies varied in different groups of patients. Patients that received long-term, high level care or involuntary treatments reported more negative SDM dyadic deviation values than other patients. To address this problem, therapists should increase flexibility in the decision processes and facilitate a psychological compensation for strict frameworks, focusing on treatment aspects where real choices exist. Patients that report more negative SDM dyadic deviation values consume a large proportion of mental health care resources. Therefore, initiatives to assimilate their perspectives into decision situations are likely to optimize the treatment courses.

## Conclusion

Patients that required high levels of care, such as in-patient and day-care treatments, involuntary treatments, and prolonged treatments (more than 2 years), had a higher probability of reporting lower SDM scores than their therapists. Identification of these patient groups might facilitate the implementation of targeted service efforts to improve SDM and achieve better treatment outcomes.

## Data Availability Statement

The datasets generated for this study are available on request to the corresponding author.

## Ethics Statement

The studies involving human participants were reviewed and approved by the Norwegian Regional Committee for Research Ethics (No. 2016/1781). The patients/participants provided their written informed consent to participate in this study.

## Author Contributions

All authors contributed to planning and conducting the study, registration and quality check of data, interpretation of data, and drafting and writing the final manuscript. KS significantly contributed in the process of writing the article, and reviewed and commented on the analyses.

## Conflict of Interest

The authors declare that the research was conducted in the absence of any commercial or financial relationships that could be construed as a potential conflict of interest.
